# Neurodegeneration in diabetic retinopathy: does it really matter?

**DOI:** 10.1007/s00125-018-4692-1

**Published:** 2018-07-20

**Authors:** Rafael Simó, Alan W. Stitt, Thomas W. Gardner

**Affiliations:** 1 Diabetes and Metabolism Research Unit, Vall d’Hebron Research Institute, Centro de Investigación Biomédica en Red de Diabetes y Enfermedades Metabólicas Asociadas (CIBERDEM), Instituto de Salud Carlos III (ISCIII), Universitat Autònoma de Barcelona, Pg. Vall d’Hebron 119-129, 08035 Barcelona, Spain; 2 Centre for Experimental Medicine, School of Medicine, Dentistry and Biomedical Sciences, Queen’s University, Belfast, UK; 3 Department of Ophthalmology and Visual Sciences, Kellogg Eye Center, University of Michigan Medical School, Ann Arbor, MI USA

**Keywords:** Diabetic retinopathy, Microvascular impairment, Neurodegeneration, Neuroprotection, Neurovascular unit, Personalised medicine, Review

## Abstract

The concept of diabetic retinopathy as a microvascular disease has evolved, in that it is now considered a more complex diabetic complication in which neurodegeneration plays a significant role. In this article we provide a critical overview of the role of microvascular abnormalities and neurodegeneration in the pathogenesis of diabetic retinopathy. A special emphasis is placed on the pathophysiology of the neurovascular unit (NVU), including the contributions of microvascular and neural elements. The potential mechanisms linking retinal neurodegeneration and early microvascular impairment, and the effects of neuroprotective drugs are summarised. Additionally, we discuss how the assessment of retinal neurodegeneration could be an important index of cognitive status, thus helping to identify individuals at risk of dementia, which will impact on current procedures for diabetes management. We conclude that glial, neural and microvascular dysfunction are interdependent and essential for the development of diabetic retinopathy. Despite this intricate relationship, retinal neurodegeneration is a critical endpoint and neuroprotection, itself, can be considered a therapeutic target, independently of its potential impact on microvascular disease. In addition, interventional studies targeting pathogenic pathways that impact the NVU are needed. Findings from these studies will be crucial, not only for increasing our understanding of diabetic retinopathy, but also to help to implement a timely and efficient personalised medicine approach for treating this diabetic complication.

**Electronic supplementary material:**

The online version of this article (10.1007/s00125-018-4692-1) contains a slideset of the figures for download, which is available to authorised users.

## Introduction

Diabetic retinopathy is the most common complication of diabetes and remains the leading cause of preventable blindness among working-age individuals in most developed countries [1, 2]. Current treatments target late stages of diabetic retinopathy, when vision has already been significantly affected. Therefore, novel and more efficient preventive and interventional strategies based on a better understanding of pathogenesis of the early stages of the disease are needed.

The concept of diabetic retinopathy as a microvascular disease has evolved, in that it is now considered a more complex diabetic complication in which neurodegeneration plays a significant role [3–6]. In fact, the ADA has recently defined diabetic retinopathy as a highly tissue-specific neurovascular complication involving progressive disruption of the interdependence between multiple cell-types in the retina [7]. In this review, we provide a critical overview on the role of neurodegeneration in the pathogenesis of diabetic retinopathy and how this new knowledge changes the traditional view of the disease. A special emphasis is placed on the pathophysiology of the neurovascular unit (NVU), examining the contributions of microvascular and neural elements. We also discuss the impact of this new perspective on possible therapeutic avenues, whilst highlighting important scientific gaps to be addressed.

## Epidemiology and the associated economic burden

The global prevalence of diabetic retinopathy in the population with diabetes is around one-third and approximately one-tenth of these patients have the vision-threatening states typified by diabetic macular oedema (DMO) or proliferative diabetic retinopathy [1]. The number of people with visual impairment owing to diabetic retinopathy worldwide is rising and this represents an increasing proportion of all causes of blindness and moderate or severe vision impairment [2]. In addition, the presence of diabetic retinopathy is an independent indicator of other diabetic complications, such as diabetic nephropathy [8, 9], cardiovascular disease [10–12] and stroke [13, 14], thus increasing the risk of morbidity and mortality in individuals with type 2 diabetes.

For these reasons individuals with diabetic retinopathy represent a significant cost for healthcare systems, only part of which is due to ophthalmic care [15–17]. Therefore, strategies to prevent or delay the progression of diabetic retinopathy would lead to a decrease in its associated economic burden [17]. In addition, improved awareness of diabetic retinopathy and its assessment in at-risk individuals will make possible the earlier detection of other systemic complications of diabetes.

## The key role of the NVU in retinal physiology

**Fig. 1:**
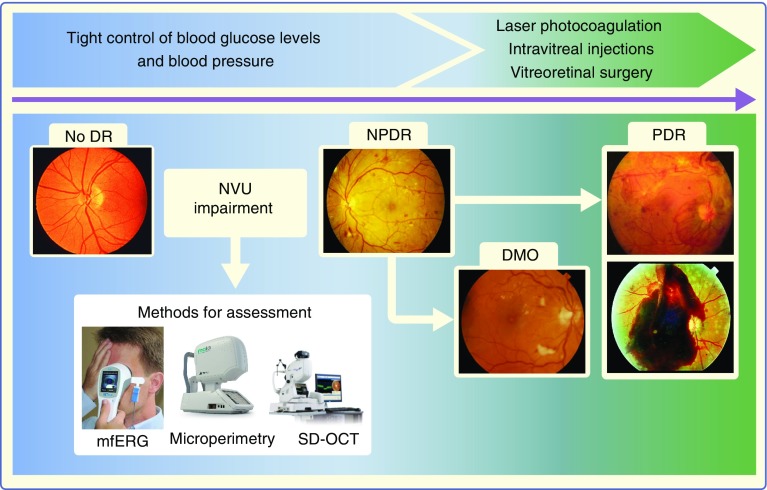
Natural history of diabetic retinopathy, based on retinal microvascular disease progression, and current treatment options. NVU impairment is an early event in the pathogenesis of diabetic retinopathy that can be assessed by functional (i.e. mfERG [with or without flickering] and microperimetry) or morphological (i.e. SD-OCT) analysis. DR, diabetic retinopathy; mfERG, multifocal electroretinogram; NPDR, non-proliferative diabetic retinopathy; PDR, proliferative diabetic retinopathy; SD-OCT, spectral domain OCT. Schematic adapted from Simó and Hernández [73] by permission from BMJ Publishing Group Limited. mfERG image, distributed under the terms of the Creative Commons Attribution-Share Alike 4.0 International License (https://creativecommons.org/licenses/by-sa/4.0/); microperimetry image, used with permission from CenterVue SpA; SD-OCT image, 3D OCT-2000, used by permission of Topcon GB Ltd. This figure is available as part of a downloadable slideset

The term ‘neurovascular unit’, was first applied to the blood–brain barrier and refers to the functional coupling and interdependency of neurons, glia and the highly specialised vasculature in the central nervous system (CNS) [18–21]. In the context of the retina, all the component cells of the NVU are in intimate communication and maintain the integrity of the inner blood–retinal barrier (iBRB) whilst dynamically regulating blood flow in response to metabolic demands. The impairment of the NVU is a primary event in the pathogenesis of diabetic retinopathy that can be examined by different methods (Fig. 1).

**Fig. 2:**
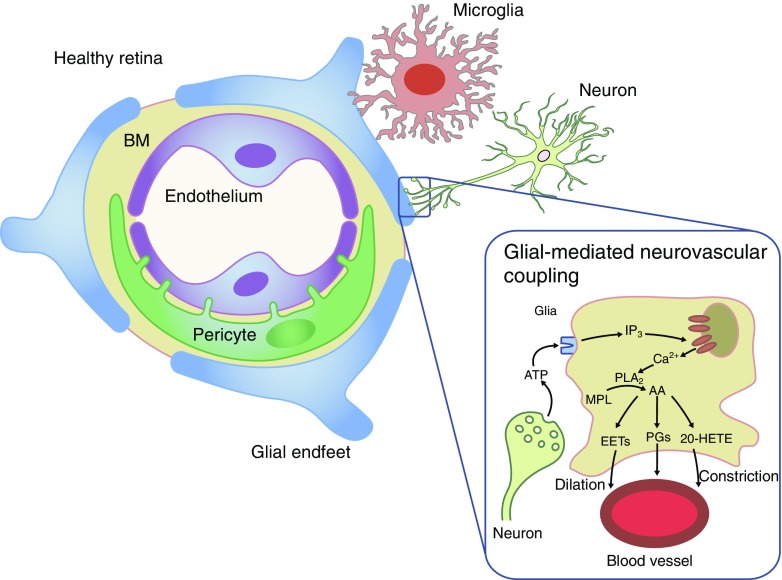
Composition of the retinal NVU. The NVU consists of vascular elements (endothelial cells, pericytes), the basement membrane (BM), glial cells (Müller cells, astrocytes), microglia and neurons. Glial-mediated neurovascular coupling is schematically represented. Synaptic release of ATP from neurons stimulates purinergic receptors on glial cells, leading to the production of inositol trisphosphate (IP_3_) and the release of Ca^2+^ from internal stores. Ca^2+^ activates phospholipase A2 (PLA_2_), which converts membrane phospholipids (MPL) to arachidonic acid (AA), which is subsequently metabolised to the vasodilators prostaglandin E_2_ (PGs) and epoxyeicosatrienoic acids (EETs), and to the vasoconstrictor 20-hydroxy-eicosatetraenoic acid (20-HETE) [23]. Interestingly, glial-induced vasodilating prostanoids are active at low NO concentrations, whereas vasoconstricting prostanoids are predominant at higher NO concentrations [24]. Healthy retina adapted from an illustration by R. Davidowitz in Duh et al [21], distributed under the terms of the Creative Commons Attribution 4.0 International License (http://creativecommons.org/licenses/by/4.0/), which permits unrestricted use, distribution, and reproduction in any medium. Glial-mediated neurovascular coupling illustration adapted from a drawing by A. Mishra in a review by Eric Newman [22], © SAGE Publications. This figure is available as part of a downloadable slideset

The components of the NVU are diverse neural cell types (i.e. ganglion cells, amacrine cells, horizontal and bipolar cells), glia (Müller cells and astrocytes), professional immune cells (microglia and perivascular macrophages) and vascular cells (endothelial cells and pericytes) [18–21] (Fig. 2). The intra-retinal vasculature lacks autonomic innervation and, therefore, a dynamic autoregulatory response of the NVU to complex circulatory and neural cues is essential to regulate blood flow through the inner retina [22, 23]. Thus, neuronal and glial-mediated neurovascular coupling is an essential normal homeostatic function of the retinal NVU.

Diabetes results in abnormal retinal blood flow, although the precise nature of this pathophysiology varies according to measurement techniques used and stage of diabetic retinopathy [22, 23]. Nevertheless, there is consistent and robust evidence that normal function of the retinal NVU is impaired in diabetes. For instance, the response to functional hyperaemia, which is critical for supplying oxygen and glucose to the active retinal neurons in the inner and middle retinal layers, becomes impaired as diabetes progresses. This response can be examined by flicker-evoked vasodilation, which is decreased in individuals in the early stages of diabetic retinopathy, even before overt signs of clinical retinopathy are observed [20–25]. These changes clearly demonstrate the relevance of neurovascular coupling or, in other words, interactions between the neurosensory retina and its blood vessels. The progressive dysfunction of neurovascular coupling may be a key causative factor in the development of clinically evident diabetic retinopathy, but longitudinal studies of retinal autoregulatory responses are needed to confirm this.

## Is microvascular disease a primary pathogenic event in the development of diabetic retinopathy?

The early stages of diabetic retinopathy include disruption of the iBRB and the thickening of the vascular basement membrane in parallel with the damage and subsequent loss of pericytes and endothelial cells.

### Blood–retinal barrier dysfunction in diabetes

The BRB consists of the iBRB and the outer BRB (oBRB). As mentioned previously, the integrity of the iBRB involves complex cell–cell communication between all the components of the NVU [26]. In contrast, the oBRB is formed by retinal pigment epithelium (RPE). In both the iBRB and the oBRB, the passage of proteins and many other macromolecules into the retina from the bloodstream is controlled by tight junctions and adherens junctions between adjacent cells (i.e. occludin, claudins and zonula occludens-1 [ZO-1]), which effectively block paracellular permeability. The disruption of the BRB, in particular the iBRB, is essential in the pathogenesis of DMO [27]. The main known contributors to the breakdown of the BRB are vascular endothelial growth factor (VEGF), proinflammatory cytokines (e.g. IL-1β, TNF-α, IL-6, monocyte chemoattractant protein-1 [MCP-1]) and components of complement. These are variously secreted from RPE, glia and immune cells. In addition, blood-circulating leukocytes may engage with adhesion molecules, such as intercellular adhesion molecule-1 (ICAM-1), vascular cell adhesion molecule (VCAM) and selectins, on the surface of endothelial cells, and the adherence of these cells to the endothelial wall may result in the occlusion of capillaries (leukostasis). Such vascular–immune cell interactions contribute to microvascular damage by releasing cytokines and superoxide via respiratory burst, which alters the integrity of the NVU [28, 29]. In the advanced stages of diabetic retinopathy, in which immune privilege is compromised, circulating immune cells and serum proteins may infiltrate the retina and vitreous, thus participating in chronic inflammation and retinal vascular and neuronal damage [30].

There is robust clinical evidence that the development and progression of retinal microvascular disease is related to glycaemic control and hypertension [6]. However, clinical information on the relationship between glycaemic control and hypertension and retinal neurodegeneration is not available. It is worth mentioning, however, that the major components of the renin–angiotensin system (RAS) have been identified in ocular tissues and they are overexpressed in the retina of individuals with diabetes [31]. In addition, the blockade of RAS in experimental models of diabetes attenuates retinal neurodegeneration [32–34]. Regarding glycaemic control, it should be noted that accumulation of advanced glycation and lipoxidation end-products, and upregulation of the receptor for advanced glycation end-products (RAGE), plays a key role in the hyperglycaemia-induced activation of Müller cells and downstream cytokine production that may contribute to diabetic retinopathy [6].

The text box ‘BRB disruption and diabetic retinopathy’ summarises the main structural factors involved in the disruption of the BRB.
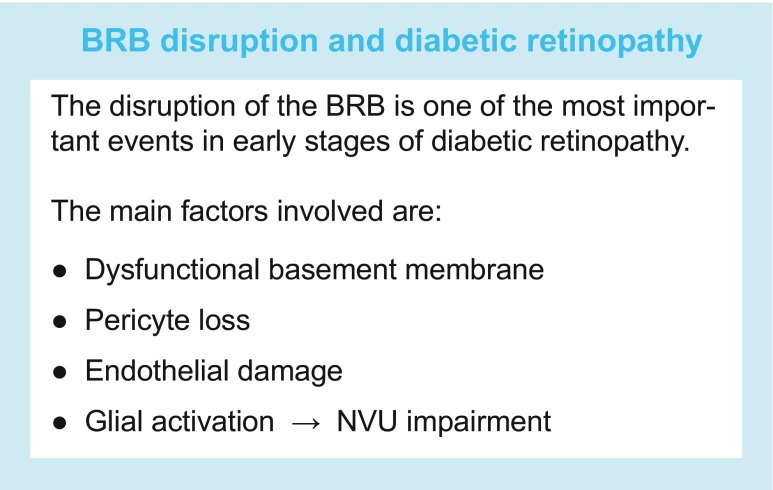


#### Basement-membrane thickening in retinal blood vessels

The vascular basement membrane is a key component of the NVU and is essential for both structural integrity and cell–matrix interactions [35]. Thickening of the capillary basement membrane is an early histological change in the retinal blood vessels in diabetic retinopathy. It is a consequence of increased synthesis of vascular basement-membrane components, such as collagen IV and laminin, in combination with reduced degradation by catabolic enzymes. These changes impair cell–cell communications, such as those that occur between endothelial cells and pericytes [6, 35]. Since the protein composition of the thickened basement membrane is modified, the charge selectivity properties of the membrane are also altered and the capacity for cell interactions that promote normal function and survival of the NVU are diminished. In addition, the thickened basement membrane acts less efficiently as a barrier, thus favouring vascular leakage [36].

#### Pericyte and endothelial cell death in diabetic retina

Pericytes are specialised contractile cells of neural crest, mesodermal and bone marrow origins; they regulate vascular tone and perfusion pressure [37]. Studies in experimental models of diabetic retinopathy have shown that pericyte dropout occurs before endothelial cell loss [38]. The loss of pericytes compromises capillary integrity leading to weakening of the iBRB and vascular leakage. The underlying mechanisms of pericyte loss during diabetic retinopathy remain to be fully elucidated and, although they have intimate physical and paracrine interactions with the vascular endothelium, demise of both pericyte and endothelial cells may occur via independent mechanisms [6, 39].

Endothelial cell injury by exposure to the diabetic milieu is a crucial event in diabetic retinopathy. When endothelial cells die, retinal capillaries become acellular. This so-called vasodegeneration or vasoregression is a central pathogenic response to chronic hyperglycaemia and initiates the progressive ischaemia characteristic of diabetic retinopathy. The importance of vasoregression in the setting of diabetic retinopathy has been comprehensively reviewed by Hammes et al [39, 40] and is conceptually divided into sequential steps: branch selection by flow dichotomy; vessel constriction; occlusion; endothelial retraction/apoptosis/reintegration; and resolution of the remaining empty vascular basement-membrane tube. Blood flow is a critical determinant of endothelial cell damage and sustained, abnormal autoregulatory responses are likely to significantly contribute to vasoregression. This is a highly complex system in which several inter-related signalling pathways are involved following the paracrine exchange of growth-factor signals between cells and differential receptor activation. There is accumulating evidence that the balance of wingless-related integration site (Wnt), Notch and angiopoietin–Tie-1 receptor signalling govern vessel formation and regression in the retina [41].

### Imaging early stages of microvascular disease: the role of optical coherence tomography angiography

**Fig. 3:**
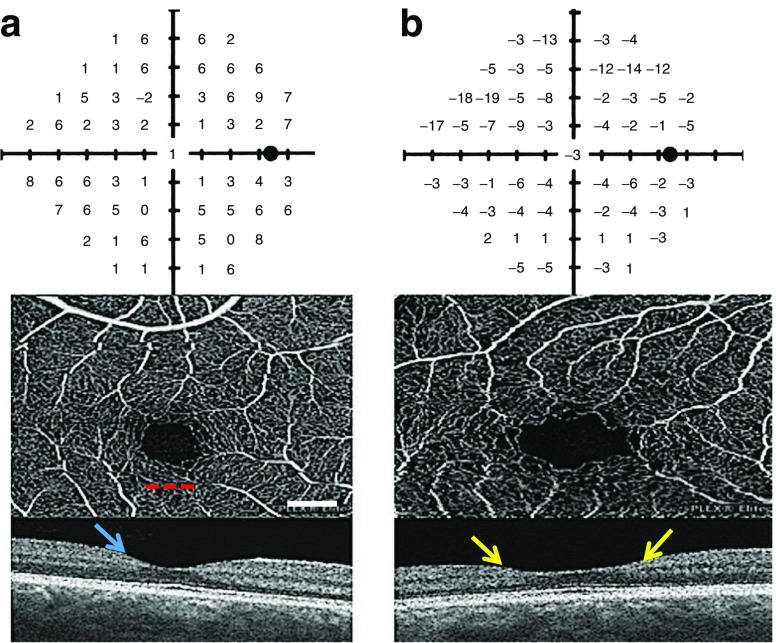
Frequency-doubling perimetry field tests with corresponding OCT angiograms and macular OCT (**a**) for a healthy 58 year old woman with 20/16 visual acuity and (**b**) for a 62 year old woman with an 18 year history of type 2 diabetes, 20/25 visual acuity and gastroparesis, but with a normal clinical examination and fluorescein angiogram (data not shown). The individual with diabetes has reduced frequency-doubling perimetry (FDP) sensitivity confirmed by repeat testing, an enlarged and irregular foveal avascular zone, a wide fovea and generalised inner retinal thinning compared with the control individual (T. W. Gardner and A. Omari, unpublished data). The red dotted line underlies the foveal avascular zone. The normal foveal depression is denoted by the blue arrow. The two yellow arrows denote inner retinal thinning. Scale bar, 0.5 mm. This figure is available as part of a downloadable slideset

Optical coherence tomography angiography (OCTA) provides depth-resolved images of blood flow in the retina with levels of detail far exceeding that obtained with older forms of imaging. Using this approach, the retinal layers can be readily visualised and the distinct capillary plexi readily imaged. OCTA provides the ability to reconstruct and view the retinal vasculature in 3D, as well as to evaluate independently the changes that occur in the superficial, intermediate and deep capillary plexi. OCTA has enabled spatial and temporal visualisation of many of the vascular changes in individuals with diabetes, such as the development of microaneurysms and loss of vascular perfusion (capillary dropout) [42]. It has revealed that such changes happen sooner and are more severe in the deep capillary plexus than in the superficial capillary layer [43]. Indeed, with use of OCTA, these microvascular alterations can be detected in patients with diabetes without clinically detectable diabetic retinopathy on fundus photography (Fig. 3).

## Is neurodegeneration the primary event in the pathogenesis of diabetic retinopathy?

A growing body of evidence clearly shows that neurodegeneration is an early event in the pathogenesis of diabetic retinopathy that could be linked to the development of microvascular abnormalities [3–6]. Therefore, the study of the underlying mechanisms leading to early disruption of the NVU and later neurodegeneration is essential for the development of new therapeutic strategies.

**Fig. 4:**
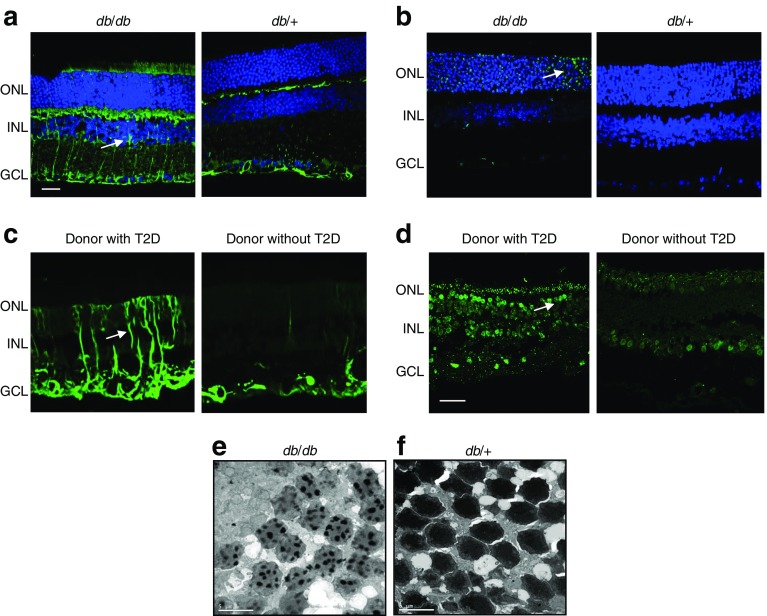
Main features of neurodegeneration: glial activation (also known as reactive gliosis) and neural apoptosis. (**a**, **c**) Glial activation (green), assessed by analysis of glial fibrillary acidic protein (GFAP) expression, and (**b**, **d**) neural apoptosis, analysed using TUNEL assay in retinas from (**a**, **b**) an experimental model of type 2 diabetes (*db*/*db* mouse) and a control (*db*/+) mouse and (**c**, **d**) human diabetic and non-diabetic donors. (**e**) Image obtained by transmission electron microscopy showing DNA fragmentation in photoreceptors in *db*/*db* mice, which is characteristic of the apoptotic process. The nuclei of cells are stained in blue. The arrows indicate glial activation (**a**, **c**) and apoptotic cells (**b**, **d**). (**a–d**) Scale bar, 20 μm; (**e**) scale bar, 5μm. GCL, ganglion cell layer; INL, inner nuclear layer; ONL, outer nuclear layer; T2D, type 2 diabetes. (**a**, **b**, **d**, **e**), images from R. Simó’s laboratory, not previously published; (**c**) Adapted from Carrasco et al [86], distributed under the terms of the Creative Commons Attribution-NonCommercial-NoDerivatives License 3.0 (http://creativecommons.org/licenses/by-nc-nd/3.0/). This figure is available as part of a downloadable slideset

The hallmarks of diabetes-induced neuroglial degeneration, which include reactive gliosis, diminished retinal neuronal function and neural-cell apoptosis, have been observed to occur before overt microangiopathy in experimental models of diabetic retinopathy and in the retina of diabetic donors [44–46] (Fig. 4). Retinal ganglion cells and amacrine cells are the first neurons in which diabetes-induced apoptosis is detected, but photoreceptors also have an increased apoptotic rate. The structural consequence of this apoptotic death is a reduced thickness of inner retinal layers and the nerve fibre layer, which can be detected by optical coherence tomography (OCT). Multifocal electroretinography (mfERG), the gold standard for assessing retinal functional impairment, has revealed that the functional repercussions of neurodegeneration consist of a delayed P1 implicit time and reduced of traces [47]. These structural and functional alterations have clinical implications in terms of deficiencies in sensory capacity, including decreased hue discrimination, contrast sensitivity, delayed dark adaptation and abnormal visual fields, and thus result in reduced vision-related quality of life [48–50].

At present, it is unknown whether neural-cell apoptosis or reactive gliosis is first in the neurodegenerative process that occurs in the retina in diabetes. However, reactive gliosis (glial activation) may play a role in damage to retinal neurons and may link the neurodegenerative process with microvascular disease. Indeed, the astrocytes and Müller cells of the NVU play a critical homeostatic function by regulating retinal blood flow, and water balance in the neural parenchyma, and by maintaining barrier function [51]. Specifically, Müller cells can undergo reactive gliosis, which is discernible by upregulation of glial fibrillary acidic protein (GFAP). Gliosis is associated with increased expression of VEGF and innate immune-related pathways, resulting in overexpression of proinflammatory cytokines and BRB dysfunction.

In addition to macroglial cells, activated microglia, the resident immune cells of the retina and infiltrating monocytes can also mediate diabetes-induced subclinical inflammation. Microglial activation is accompanied by a phenotypic change toward an ameboid shape and presents two opposite roles, triggering either proinflammatory (M1) or anti-inflammatory (M2) actions [52, 53]. In the early stages of diabetic retinopathy, the M2 response occurs concurrently with the M1 response and ameliorates inflammation and delays the progression of the disease. However, during the progression of diabetic retinopathy, the M1 response is maintained whereas the M2 response declines and the classical proinflammatory signalling pathways are chronically activated [53]. In fact, a shift from pro-survival to pro-neurotoxicity occurs, and transcriptional changes in activated microglia, mediated via the NFκB and extracellular signal-regulated kinase (ERK) signalling pathways, result in the release of various proinflammatory cytokines, chemokines, caspases and glutamate [54]. These molecular mediators contribute to disruption of the BRB and NVU impairment, and to neuronal death.

### Mechanisms linking retinal neurodegeneration and early microvascular impairment

**Fig. 5:**
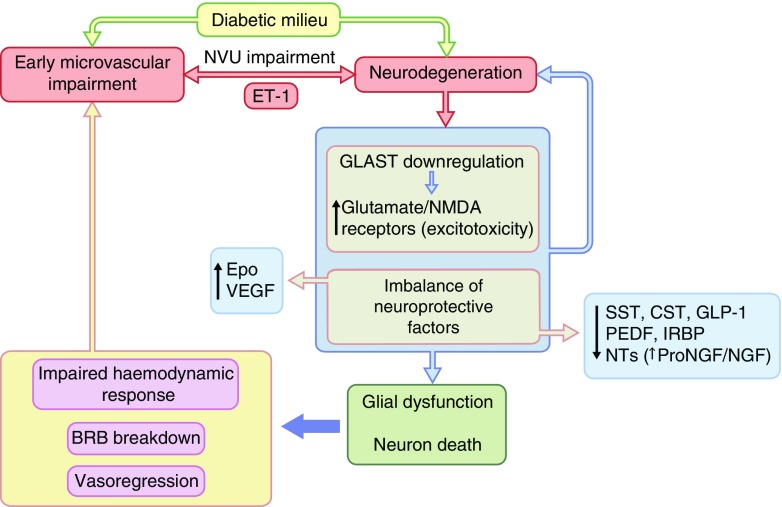
Main mechanisms involved in the link between retinal neurodegeneration and microvascular impairment in diabetes. Impaired cell–cell interaction within the NVU is critical in the pathogenesis of early stages of diabetic retinopathy. For example, ET-1 (levels of which are altered in diabetes) has a significant role in inducing both vasoconstriction and neurodegeneration via activation of the endothelin receptors ET_A_ and ET_B_, respectively. In addition, increased extracellular concentrations of glutamate, owing to downregulation of the glutamate aspartate transporter (GLAST) in those with diabetes, results in excitotoxicity and neuron death. The subsequent progressive imbalance between neuroprotective factors (somatostatin, cortistatin, glucagon-like peptide 1, PEDF etc) is a major factor in neural apoptosis and glial activation in diabetes. In terms of vascular impairment, this leads to an altered haemodynamic response, BRB breakdown and vasoregression. CST, cortistatin; Epo, erythropoietin; IRBP, interphotoreceptor retinoid-binding protein; NGF, nerve growth factor; NMDA, *N*-methyl-d-aspartate; NT, neurotrophin; ProNGF, nerve growth factor precursor; SST, somatostatin. Adapted from Simó and Hernández [4], with permission from Elsevier. This figure is available as part of a downloadable slideset

The potential mechanisms linking retinal neurodegeneration and early microvascular impairment are summarised in Fig. 5. Apart from glial-mediated vascular damage, the balance between upregulated and downregulated neuroprotective factors in the diabetic retina is very important for the fate of the retinal neurons. In early stages of diabetic retinopathy, downregulation of key factors such as pigment epithelium-derived factor (PEDF), somatostatin, glucagon-like peptide 1 (GLP-1) and other neurotrophic factors is counterbalanced by an upregulation of VEGF and erythropoietin [4, 5]. However, the downregulation of neuroprotective factors may predominate, thus contributing to retinal neurodegeneration. This finding has important therapeutic implications. In this regard, neuroprotective effects have been reported by using insulin [55], PEDF [56, 57], somatostatin [58], GLP-1 [59, 60], dipeptidyl peptidase-IV (DPP-IV) inhibitors [61] and erythropoietin or erythropoietin-linked analogues [62, 63] in various experimental models. The European Consortium for the Early Treatment of Diabetic Retinopathy (EUROCONDOR) clinical trial has recently shown that topical administration of somatostatin arrested the progression of neurodysfunction as assessed by mfERG (implicit time) in participants with some degree of neurodysfunction at baseline [64]. As an alternative target, endothelin-1 (ET-1) is upregulated in the retina in diabetes [65] and has dual deleterious action on microvessels and neurons. This is because of its capacity to bind to endothelin receptors A (ET_A_) which mainly mediates vasoconstriction and vasoregression [66], and B (ET_B_), involved in retinal neurodegeneration [67, 68]. Therefore, the blockade of ET-1 may prevent both microvascular disease and neurodegeneration induced by diabetes.

It should be noted that retinal neurons, themselves, including photoreceptors, may be an important source of oxidative stress that help drive the proinflammatory environment in diabetic retinopathy [69, 70], thus leading to vasoregression. In addition, it has recently been reported that photoreceptor cells release inflammatory products, which directly contribute to increased retinal endothelial permeability in mouse models of diabetes [71]. Furthermore, retinal neuronal cells may secrete molecules, such as semaphorin-3A, that promote BRB dysfunction, and may contribute to macular oedema [72].

When assessed by electroretinogram or other electrophysiological and psychophysical methods, impaired retinal function has been found to occur in individuals with diabetes who do not have detectable microvascular abnormalities [73]. In this regard, neuroretinal dysfunction, as assessed by mfERG, has been reported in individuals with type 1 diabetes without BRB leakage, the latter measured by vitreous fluorometry [74]. In addition, progressive thinning of the inner retina over time (assessed by OCT) occurs in murine experimental models [75], and in humans with type 1 diabetes, without any associated reduction of capillary density [76]. Furthermore, though only performed in a small number of individuals, prospective studies using mfERG have shown that increased implicit time can predict the development of visible vascular abnormalities over a 1 to 3 year period [77, 78]. However, baseline fluorescein angiograms were not performed in these studies, so it is possible that subclinical lesions existed at study entry. These findings raise the possibility, but do not prove, that retinal neurodegeneration may precede the onset of diabetes-induced vascular changes. Robust observational studies or interventional clinical trials that examine the neuro–vascular relationships are lacking. The recent randomised, placebo-controlled, Phase II–III EUROCONDOR study failed to show any effect of two neuroprotective drugs (brimonidine and somatostatin; administered by eye drops) in preventing or arresting microvascular disease [64]. Nevertheless, it should be noted that the short follow-up of this clinical trial (2 years), the inclusion of a high proportion of patients with no or very mild microvascular disease, and the excellent metabolic control during follow-up (mean HbA_1c_, 54.1 mmol/mol [7.1%] in all arms) could explain the negative findings with regards to the effects of neuroprotection on the development or progression of retinal microvascular disease.

## How do we integrate the microvascular and neural components?

In order to integrate retinal microangiopathy and neuropathy into the definition of eye disease in diabetes, the term ‘diabetic retinal disease’, instead of ‘diabetic retinopathy’, has been proposed for a more comprehensive definition of the disease [79]. However, our knowledge of the relationship between diabetes-induced retinal neurodegeneration and microvascular disease is still limited. To our knowledge, there are no published studies in which cell-specific defects in microvascular or neural cells result in a diabetic retinopathy-like phenotype. In addition, although several studies using neuroprotective drugs, such GLP-1 and DPP-IV inhibitors, have prevented vascular leakage in rodents [59–61], it is unknown if this effect was due to neuroprotection or to direct vascular effects. In addition, recent results from the EUROCONDOR study showed that a significant proportion of individuals with type 2 diabetes present with early microvascular disease without detectable neurodysfunction [64, 80]. Therefore, it seems that neurodegeneration is not always the apparent primary event in the natural history of diabetic retinopathy. In this regard, it is possible that neurodegeneration could herald diabetic retinopathy in some subsets of patients, but neurodegeneration and microvascular disease could occur independently in others. This new comprehensive understanding of diabetic retinopathy emphasises the need for better phenotyping and stratification of patients with diabetic retinopathy, not only by use of the methods addressed to measure microvascular impairment (e.g. OCTA), but also by incorporating measurements of retinal function.

The relative sensitivity of the methods used to assess neurodysfunction and microvascular damage should be taken into account when examining whether neuronal or vascular dysfunction occur first. In this regard, prospective studies using new technologies (e.g. fundus microperimetry, OCTA and OCT-based oximetry) are needed.

It should be noted that diabetic retinopathy may be a common response to multiple metabolic injuries that depend on the duration and severity of diabetes, and are modulated by the presence of hypertension, dyslipidaemia, systemic inflammation and renal disease. Indeed, the strikingly similar phenotypic appearance of diabetic and radiation retinopathy suggests that the retina has a limited response pattern to a variety of insults.

In view of consistent evidence from studies into both experimental models and humans, that neurodegeneration is an early event in the retina in diabetes, it could be hypothesised that glial activation and some degree of neural apoptosis exists in the retina of most individuals with long-term diabetes. However, it is apparent that only a subset of these individuals will develop microvascular disease, which could initially be triggered by glial activation and neurodegeneration. However, in later stages these two pathophysiological events may evolve independently.

## Retinal neurodegeneration is a biomarker of neurodegenerative diseases

Numerous epidemiological studies have demonstrated that individuals with type 2 diabetes have a significantly higher risk of developing neurodegenerative diseases, in particular, Alzheimer’s disease [81]. The retina is ontogenically brain-derived tissue, so it may provide an easily accessible and non-invasive way of examining CNS pathology. Therefore, it could be postulated that, in individuals who develop brain neurodegeneration, a neurodegenerative process co-occurs in the retina (‘the eye as a window of the brain’). In fact, both diabetes-induced retinal neurodegeneration and Alzheimer’s disease share several pathogenic pathways, such as insulin signalling impairment, low-grade inflammation, the accumulation of advanced glycation end-products (AGEs) and an increase in oxidative stress [81]. In addition, several pathogenic pathways triggered in the brain of those with neurodegenerative diseases have also been found to be triggered in the retinas of individuals with type 2 diabetes [82].

Current neuroimaging modalities, such as MRI, may not be able to detect subtle subclinical changes in the brain (resolution <100–500 μm), so advances in retinal imaging (i.e. OCT) may provide an additional tool for new and potentially important insights into neurodegenerative processes [83]. Functional assessment of the retina could also be used as an indirect method to explore events in the brain. In a recent prospective study, retinal sensitivity assessed by microperimetry significantly correlated with variables related to brain neurodegeneration and cognitive status [84]. This pilot study suggested that the assessment of retinal sensitivity by microperimetry could be a useful biomarker for identifying individuals with type 2 diabetes who are at risk of developing Alzheimer’s disease. This is an important issue since unrecognised cognitive dysfunction can affect treatment adherence and diabetes self-management, resulting in poor glycaemic control, an increased frequency of severe hypoglycaemic episodes and increased hospital admissions [81]. For these reasons, the early diagnosis of cognitive impairment is not only recommendable in itself, but also permits a more personalised treatment approach for patients with type 2 diabetes. In this regard, it should be noted that the ADA recommends individualised diabetes treatment, taking into account the cognitive capacity of patients [85].
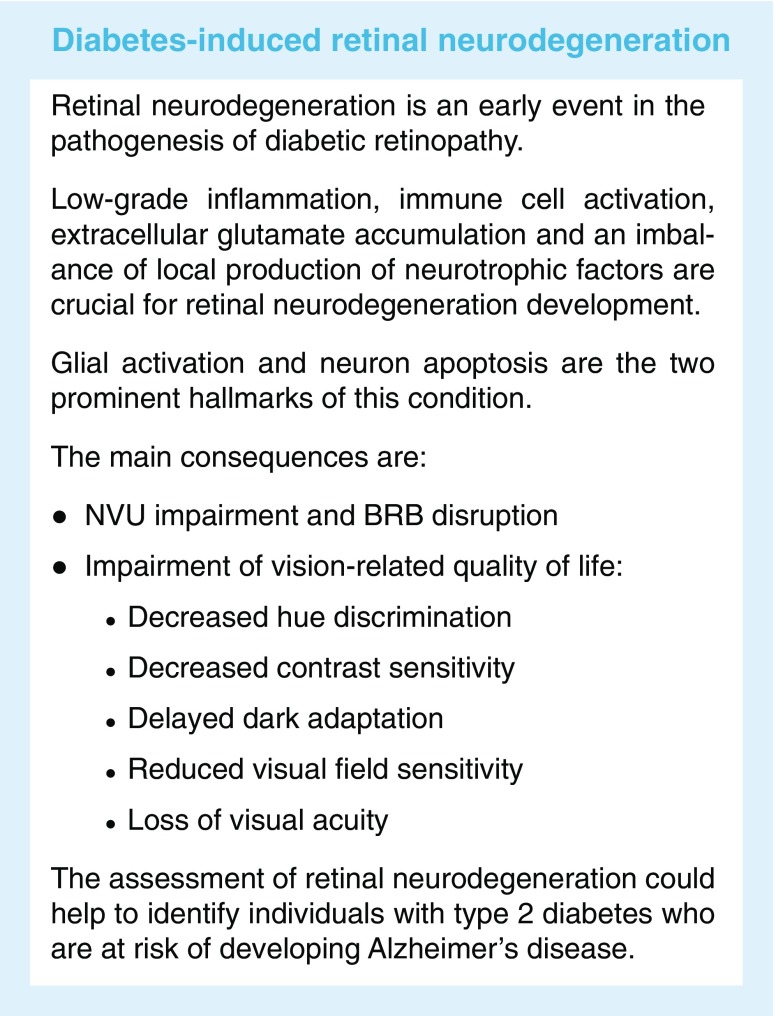


## Where do we go from here?

Diabetes causes not only classical retinal microangiopathy and DMO, but also neurodegeneration, and these events coalesce with progressive disruption of the retinal NVU. Glial dysfunction plays a crucial role in diabetes-induced neurovascular coupling impairment, thus contributing to the early stages of microvascular disease. However, our knowledge regarding the cellular and molecular mechanisms that link retinal neurodegeneration and microvascular disease remains limited and more research is needed to understand the complex intercellular dynamics within the NVU in health and diabetes. Current evidence suggests that neurodegeneration is an early event in diabetic retinopathy but may or may not be related to the development and progression of microvascular disease. This should be tested further in future long-term clinical trials using highly sensitive new technologies combined with improved stratification of participants.

The consequences of progressive retinal diabetic neurodegeneration, specifically in the absence of clinically appreciable diabetic retinopathy, have been gaining attention [79]. In this regard, it is notable that the loss of neuroretinal thickness (nerve fibre layer, ganglion cell layer and inner plexiform layer) in people with diabetes with no or minimal diabetic retinopathy is around 0.54 μm per year [76]. This mean a loss of 5.4 μm over 10 years and, remarkably, is equivalent to the loss found in severe glaucoma. Since this neuron loss is related to deficient sensory capacity and vision-related quality of life, periodic assessments of neurodegeneration/neurodysfunction in the diabetic population is strongly recommended. In addition, the emergent development of neuroprotective drugs to treat diabetic retinopathy points to screening for retinal neurodysfunction as critical for identifying the subset of patients in whom neuroprotective treatment might be of benefit. Additionally, the assessment of retinal neurodegeneration could be an important index of cognitive status, thus helping to identify individuals at risk of dementia.

In summary, it is now recognised that during diabetes, retinal glial, neural and microvascular dysfunction is interdependent and essential for the development of diabetic retinopathy. Despite this intricate relationship, it should be noted that retinal neurodegeneration is a critical endpoint and neuroprotection, itself, can be considered as a target, independently of its potential impact on microvascular disease. Hence, the decades-old grading schemes of diabetic retinopathy, based solely on non-quantitative assessment of microvascular abnormalities, should be replaced by robust quantitative readouts that are reflective of progressive dysfunction in the retinal NVU. In addition, more interventional studies targeting pathogenic pathways that impact the NVU and that offer both vaso- and neuroprotection are needed. This will be crucial, not only for increasing our understanding of diabetic retinopathy, but also to implement a timely and efficient personalised medicine approach for the treatment of this disease.

## Electronic supplementary material

ESM Slideset of figures (PPTX 1635 kb)

## Abbreviations

BRB: Blood–retinal barrier
CNS: Central nervous system
DMO: Diabetic macular oedema
DPP-IV: Dipeptidyl peptidase-IV
ET-1: Endothelin-1
EUROCONDOR: European Consortium for the Early Treatment of Diabetic Retinopathy
GLP-1: Glucagon-like peptide 1
iBRB: Inner blood–retinal barrier
mfERG: Multifocal electroretinogram
NVU: Neurovascular unit
oBRB: Outer blood–retinal barrier
OCT: Optical coherence tomography
OCTA: Optical coherence tomography angiography
PEDF: Pigment epithelium-derived factor
RAS: Renin–angiotensin system
RPE: Retinal pigment epithelium
VEGF: Vascular endothelial growth factor
