# Supplementary material for: Neurodegeneration in diabetic retinopathy: does it really matter?
Source: Diabetologia. 2018 Jul 20;61(9):1902–12. doi: 10.1007/s00125-018-4692-1 (PMC6096638; doi:10.1007/s00125-018-4692-1)
Supplement: Supplementary file 1 — (PPTX 1635 kb) [file 125_2018_4692_MOESM1_ESM.pptx]

## Slide 1
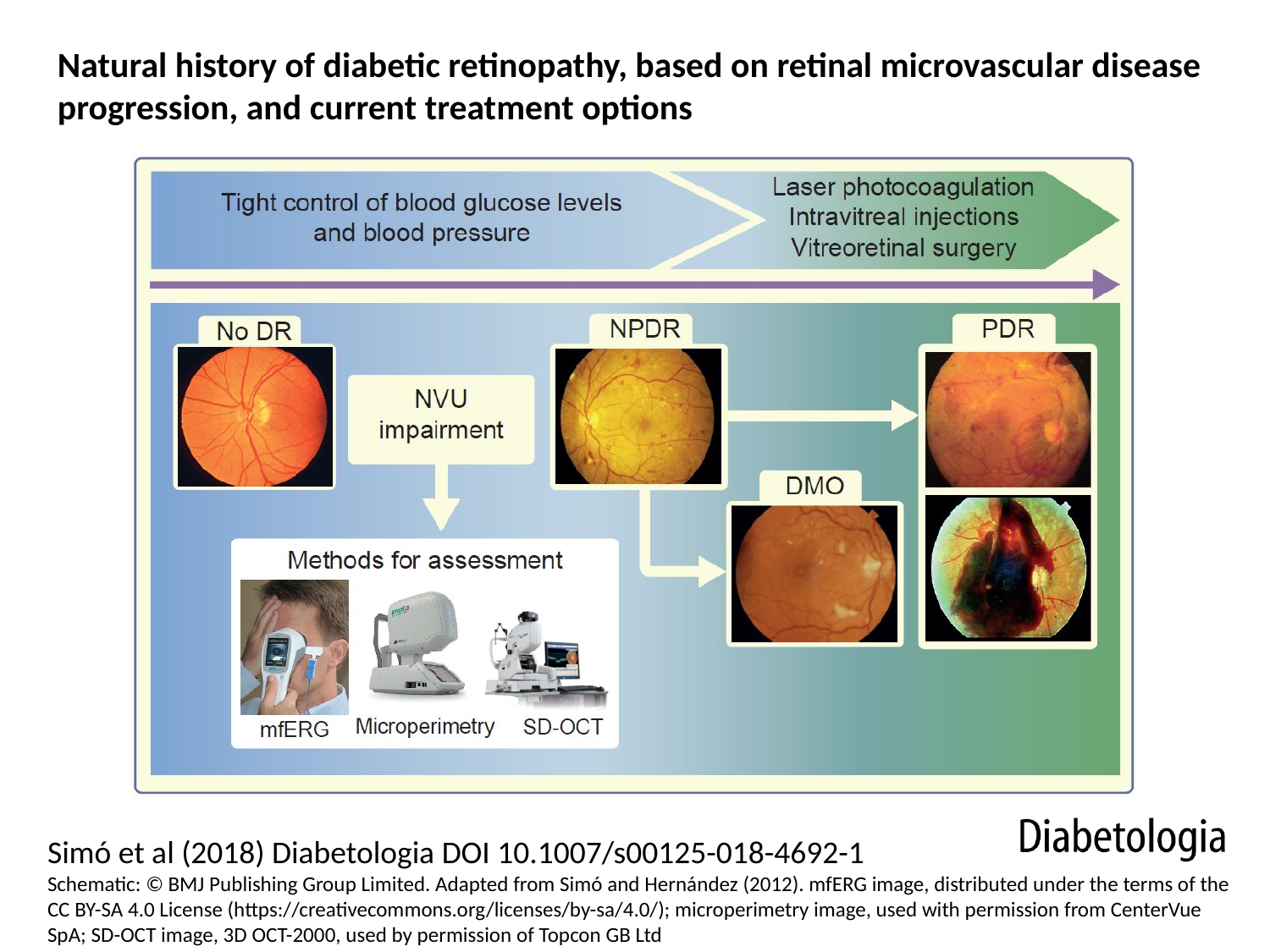

Natural history of diabetic retinopathy, based on retinal microvascular disease progression, and current treatment options
Simó et al (2018) Diabetologia DOI 10.1007/s00125-018-4692-1
Schematic: © BMJ Publishing Group Limited. Adapted from Simó and Hernández (2012). mfERG image, distributed under the terms of the CC BY-SA 4.0 License (https://creativecommons.org/licenses/by-sa/4.0/); microperimetry image, used with permission from CenterVue SpA; SD-OCT image, 3D OCT-2000, used by permission of Topcon GB Ltd

## Slide 2
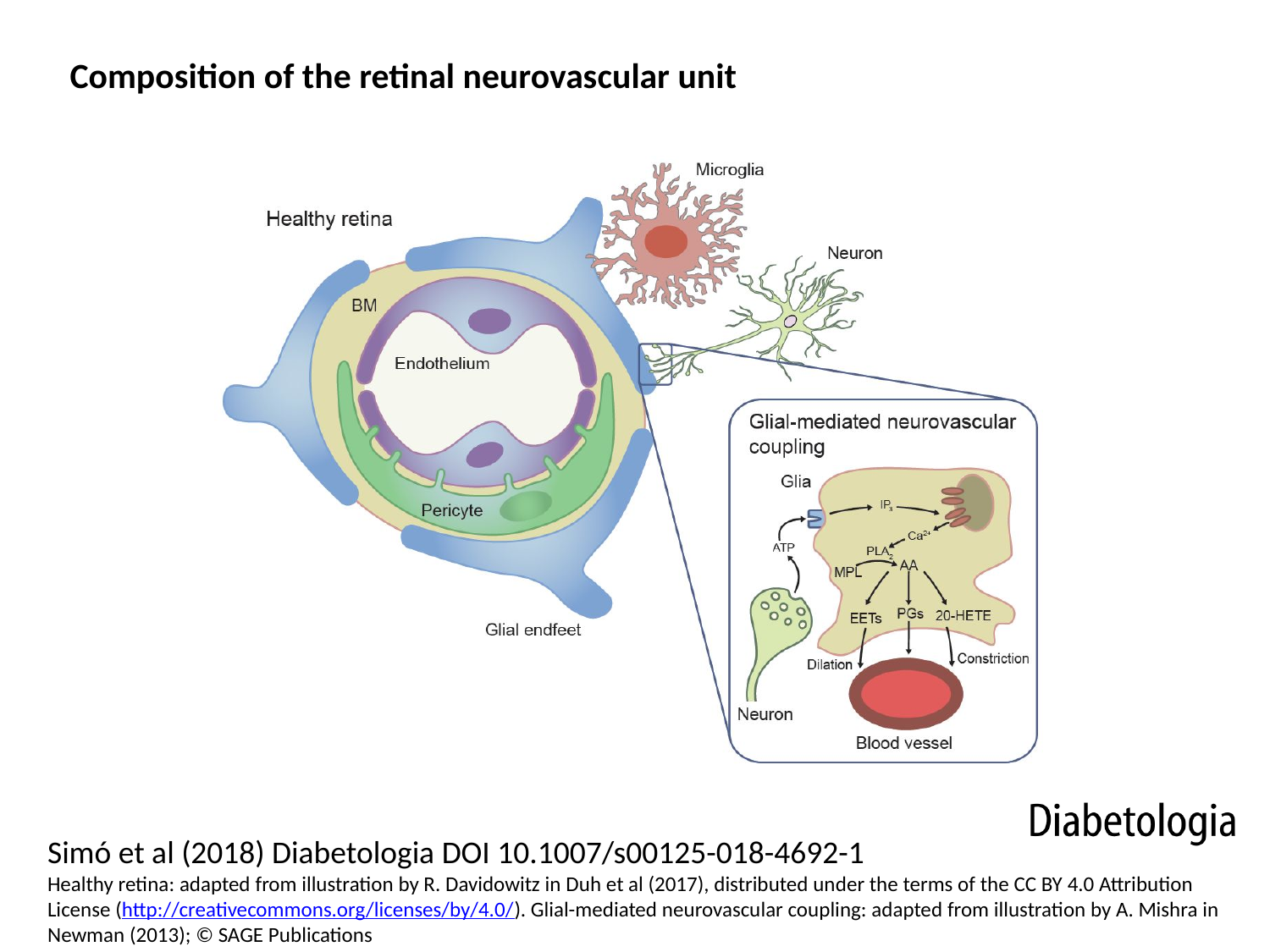

Composition of the retinal neurovascular unit
Simó et al (2018) Diabetologia DOI 10.1007/s00125-018-4692-1
Healthy retina: adapted from illustration by R. Davidowitz in Duh et al (2017), distributed under the terms of the CC BY 4.0 Attribution License (http://creativecommons.org/licenses/by/4.0/). Glial-mediated neurovascular coupling: adapted from illustration by A. Mishra in Newman (2013); © SAGE Publications

## Slide 3
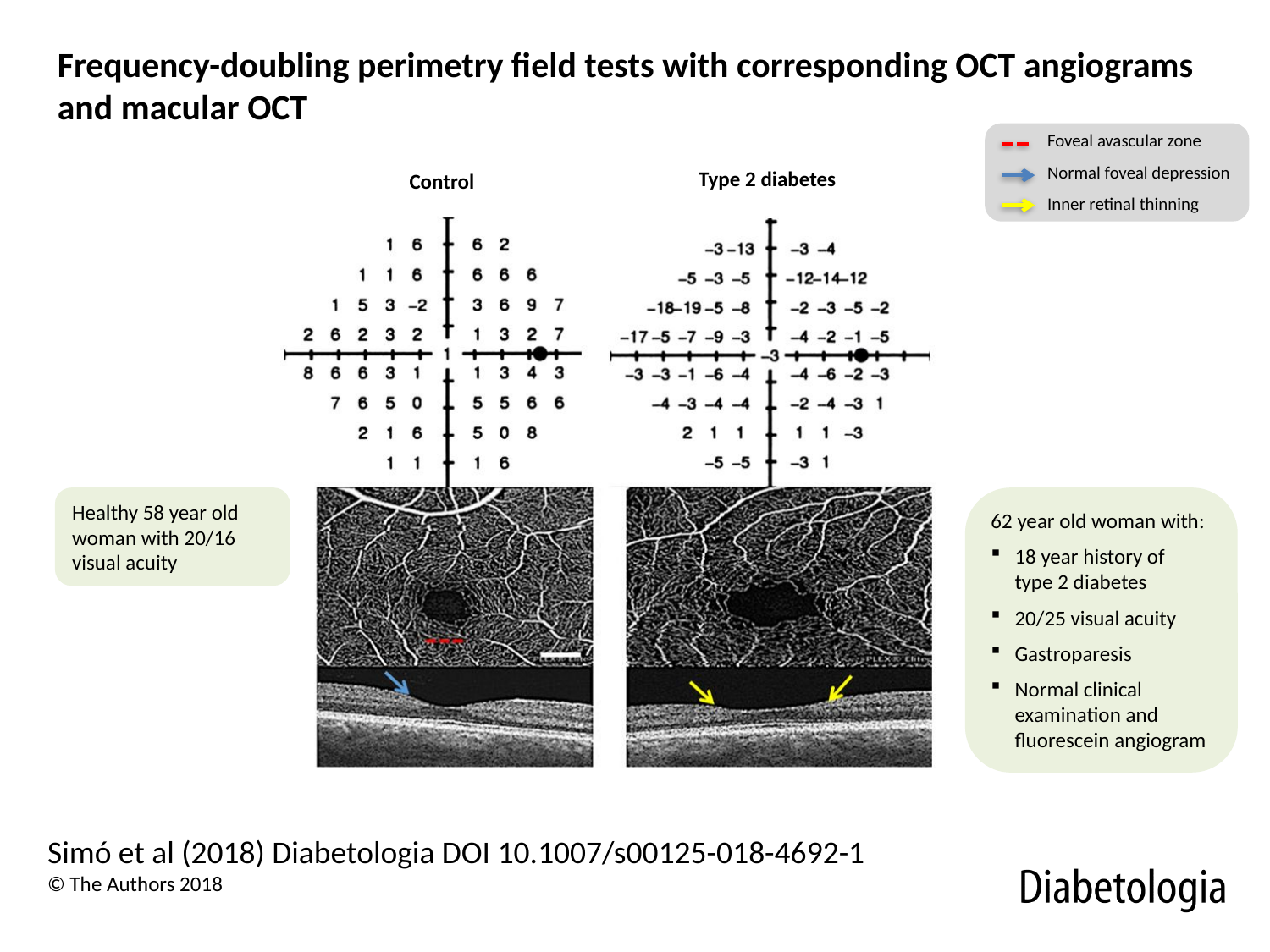

Frequency-doubling perimetry field tests with corresponding OCT angiograms and macular OCT
Foveal avascular zone
Normal foveal depression
Inner retinal thinning
Type 2 diabetes
Control
Healthy 58 year old woman with 20/16 visual acuity
62 year old woman with:
18 year history of type 2 diabetes
20/25 visual acuity
Gastroparesis
Normal clinical examination and fluorescein angiogram
Simó et al (2018) Diabetologia DOI 10.1007/s00125-018-4692-1
© The Authors 2018

## Slide 4
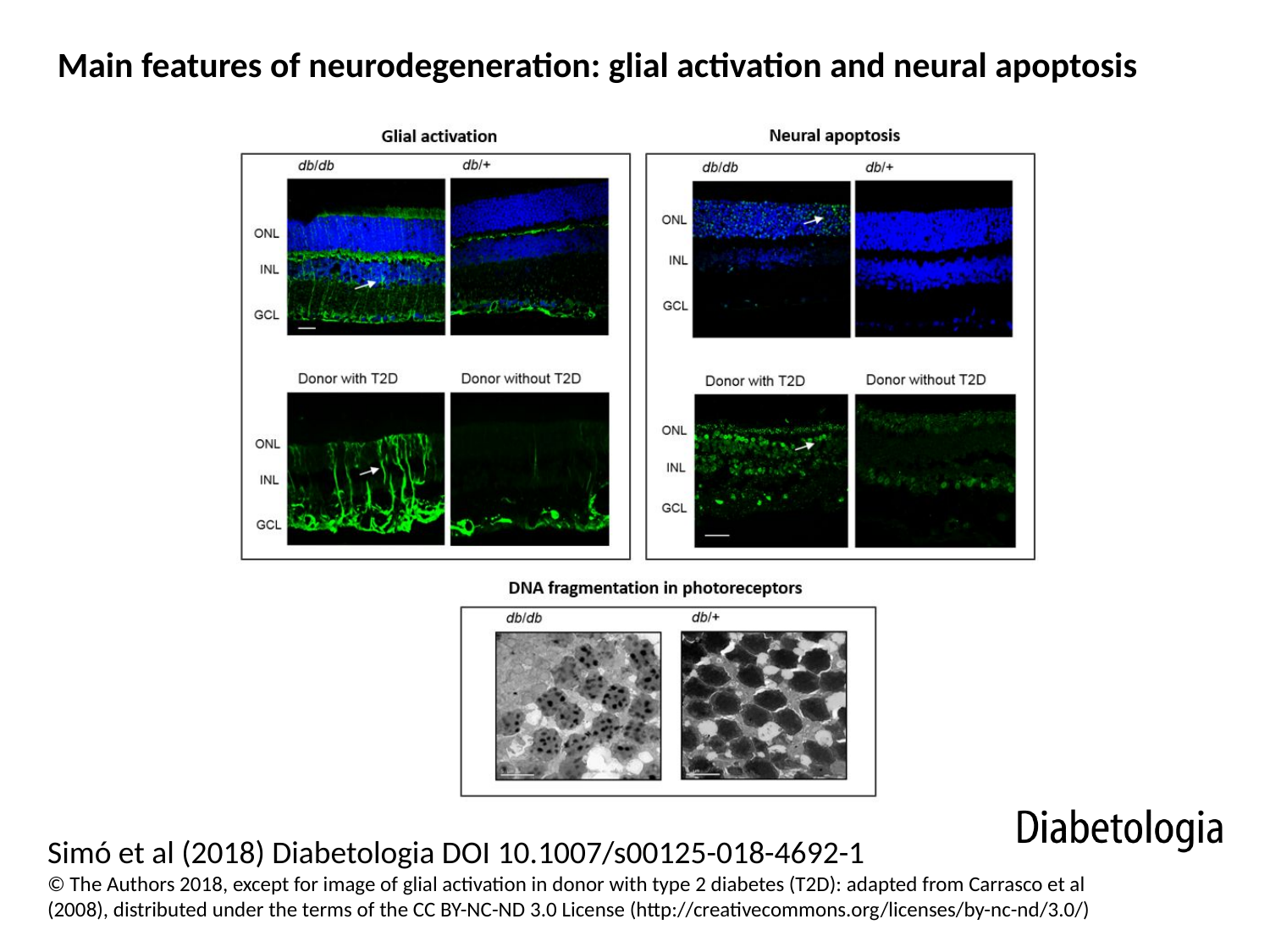

Main features of neurodegeneration: glial activation and neural apoptosis
Simó et al (2018) Diabetologia DOI 10.1007/s00125-018-4692-1
© The Authors 2018, except for image of glial activation in donor with type 2 diabetes (T2D): adapted from Carrasco et al (2008), distributed under the terms of the CC BY-NC-ND 3.0 License (http://creativecommons.org/licenses/by-nc-nd/3.0/)

## Slide 5
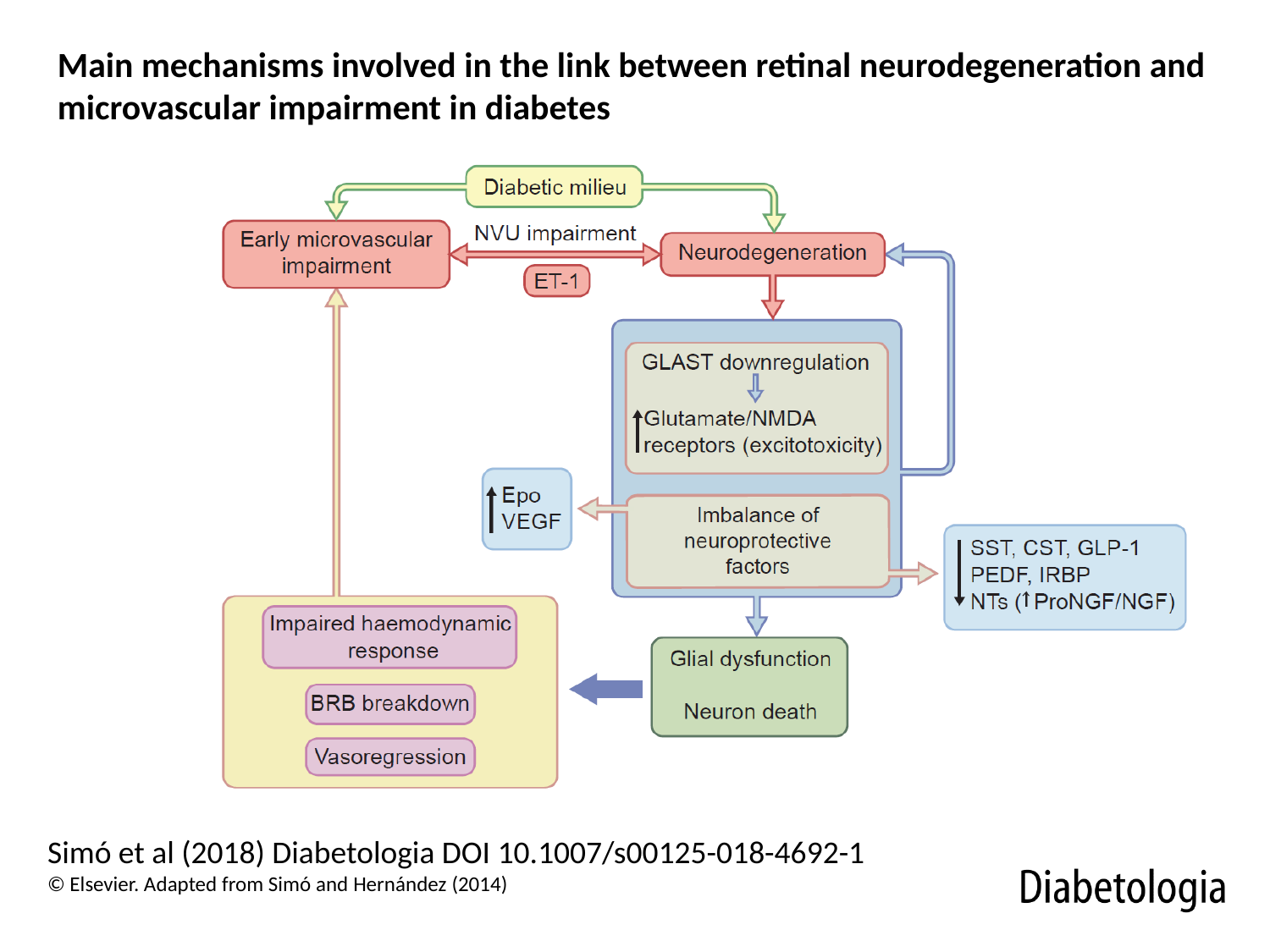

Main mechanisms involved in the link between retinal neurodegeneration and microvascular impairment in diabetes
Simó et al (2018) Diabetologia DOI 10.1007/s00125-018-4692-1
© Elsevier. Adapted from Simó and Hernández (2014)
